# Modulatory Effect of Intermittent Fasting on Adipose Tissue Inflammation: Amelioration of Cardiovascular Dysfunction in Early Metabolic Impairment

**DOI:** 10.3389/fphar.2021.626313

**Published:** 2021-04-09

**Authors:** Haneen S. Dwaib, Ibrahim AlZaim, Ali H. Eid, Omar Obeid, Ahmed F. El-Yazbi

**Affiliations:** ^1^ Department of Pharmacology and Toxicology, Faculty of Medicine, American University of Beirut, Beirut, Lebanon; ^2^ Department of Nutrition and Food Sciences, Faculty of Agricultural and Food Sciences, American University of Beirut, Beirut, Lebanon; ^3^ Department of Biochemistry and Molecular Genetics, Faculty of Medicine, American University of Beirut, Beirut, Lebanon; ^4^ Department of Basic Medical Sciences, College of Medicine, QU Health, Qatar University, Doha, Qatar; ^5^ Biomedical and Pharmaceutical Research Unit, QU Health, Qatar University, Doha, Qatar; ^6^ Department of Pharmacology and Toxicology, Faculty of Pharmacy, Alexandria University, Alexandria, Egypt; ^7^ Faculty of Pharmacy, Al-Alamein International University, Alamein, Egypt

**Keywords:** cardiometabolic syndrome, early metabolic dysfunction, prediabetes, perivascular adipose inflammation, therapeutic fasting

## Abstract

Cardiometabolic syndrome (CMS) is a cluster of maladaptive cardiovascular, renal, thrombotic, inflammatory, and metabolic disorders. It confers a high risk of cardiovascular mortality and morbidity. CMS is triggered by major shifts in lifestyle and dietary habits with increased consumption of refined, calorie-dense diets. Evidence indicates that diet-induced CMS is linked to Adipose tissue (AT) inflammation. This led to the proposal that adipose inflammation may be involved in metabolic derangements, such as insulin resistance and poor glycemic control, as well as the contribution to the inflammatory process predisposing patients to increased cardiovascular risk. Therefore, in the absence of direct pharmacological interventions for the subclinical phase of CMS, time restricted feeding regimens were anticipated to alleviate early metabolic damage and subsequent comorbidities. These regimens, referred to as intermittent fasting (IF), showed a strong positive impact on the metabolic state of obese and non-obese human subjects and animal models, positive AT remodeling in face of overnutrition and high fat diet (HFD) consumption, and improved CV outcomes. Here, we summarize the available evidence on the role of adipose inflammation in triggering cardiovascular impairment in the context of diet induced CMS with an emphasis on the involvement of perivascular adipose tissue. As well, we propose some possible molecular pathways linking intermittent fasting to the ameliorative effect on adipose inflammation and cardiovascular dysfunction under such circumstances. We highlight a number of targets, whose function changes in perivascular adipose tissue inflammation and could be modified by intermittent fasting acting as a novel approach to ameliorate the inflammatory status.

## Introduction

Cardiometabolic syndrome (CMS) has long been recognized as a cluster of maladaptive cardiovascular, renal, thrombotic, inflammatory, and metabolic disorders by several global health authorities (Castro et al., 2003). At the core of the framework of processes resulting in this syndrome lies multi-organ insulin resistance (IR), especially adipose tissue (AT) IR, that is considered key in CMS pathogenesis and prognosis (Kirk and Klein, 2009). While CMS is typically considered a pre-morbid condition, it is associated with a high risk of cardiovascular mortality and morbidity due to ischemic heart disease, ischemic stroke, cardiac metabolic dysfunction, and heart failure (Ash-Bernal and Peterson, 2006; von Bibra et al., 2016). Although recent estimates indicate a 35% surge in prevalence of CMS over the past 20 years (Moore et al., 2017), the definition and the diagnostic criteria of CMS are still debatable (Kirk and Klein, 2009). While most authorities agree on the inclusion of abnormalities of blood pressure, HDL-cholesterol, triacylglycerol and glucose tolerance, in addition to central obesity, they use different cut-off values for diagnosis and non-identical rank order of importance (Simmons et al., 2010).

Metabolic disorders that could be considered a culmination of CMS, including diet-induced obesity (DIO) (WHO, 2019) and type 2 diabetes (International Diabetes Federation, 2019), have increased in prevalence over the past few decades together with their associated cardiovascular morbidities. This steep rise could be attributed to major shifts in lifestyle and dietary habits comprising overnutrition and increased consumption of refined, calorie-dense diets rich in saturated fats and simple sugars (Lutsey et al., 2008; Misra et al., 2010). The rising health and economic burden makes an early intervention with CMS prudent. Significantly, several challenges face pre-emptive mitigation of CMS complications, particularly that cardiovascular risk increases at early disease stages that do not meet established diagnostic criteria of overt metabolic disease (Grundy, 2012). Moreover, subtle forms of vascular dysfunction, including impaired microvascular vasodilation and blood flow auto-regulation leading to cardiac consequences can occur in absence of overt signs of atherosclerosis and other vascular disease in the context of early CMS (Tune et al., 2017). Thus, a detailed study of the underlying cardiometabolic pathophysiology is warranted.

In this regard, research in the past two decades highlighted the association of chronic excessive caloric intake triggering negative adipose tissue remodeling (ATR) with the development of obesity and type 2 diabetes (Choe et al., 2016). Given the long recognized relationship between insulin resistance and the pathophysiology of CMS (Reaven, 1988), ATR and inflammation is very likely to play a central role in this pathogenesis (Richardson et al., 2013; Shimizu et al., 2013; Kohlgruber and Lynch, 2015). Indeed, increased cardiovascular risk in patients with metabolic disorders is linked to inflammation (Festa et al., 2003), possibly initiating in the AT in response to insulin resistance and hyperinsulinemia (Pedersen et al., 2015), making such a pathology an attractive target for early alleviation of CMS. On the other hand, as overnutrition and positive energy balance being the key triggers of CMS, calorie restriction becomes an attractive intervention to study as a possible way to prevent, improve and even treat CMS cardiovascular manifestations. Intermittent fasting (IF) regimens hypothesized to impact metabolic health can adopt several patterns (Patterson et al., 2015; Antoni et al., 2017). Complete alternate-day fasting involves the alternation between fasting and eating days, while time-restricted feeding involves ad libitum energy intake within defined timeframes, allowing for the establishment of regular fasting intervals. Other IF regimens include modified fasting regimens which involve the consumption of 20–25% of energetic needs on scheduled fasting days and ad libitum feeding on other days. Religious fasting can take a variety of forms, the most practiced of which is Ramadan fasting which involves a sunrise-to-sunset fast for a month period yearly. Other religious fasts involve abstinence from food consumption for prolonged periods of time that can extend for days. Importantly, the different regimens of IF have been shown to improve the metabolic state of obese and non-obese animal models and human subjects (Patterson et al., 2015; St-Onge et al., 2017). In this context, it becomes necessary to understand the potential impact of such an intervention on the pathogenetic pathways linking early metabolic dysfunction to cardiovascular impairment. As such, in the present review, we summarize the available evidence on the role of negative adipose remodeling in triggering cardiovascular impairment in the context of CMS with an emphasis on the involvement of perivascular adipose tissue (PVAT). As well, the possible molecular pathways linking IF to the ameliorative effect on adipose inflammation and cardiovascular dysfunction under such circumstances are examined.

## Adipose Tissue Remodeling in Response to Increased Caloric Intake

AT is one of the major players in the pathophysiology of CMS. Over the past two decades, our traditional view of the AT as a mere storage pool of excess calories evolved to encompass its endocrine functions as an integral factor in the regulation of glucose and lipid homeostasis (Guilherme et al., 2008). Such an endocrine function arises from complex interactions between adipocytes and the cells of the stromal vascular fraction (SVF), which modulates AT functioning in homeostatic and pathological conditions. This endocrine function is ascribed to white adipose tissue (WAT), which comprises unilocular adipocytes that specialize in energy storage and production of several crucial protein factors collectively called adipokines. Brown adipose tissue (BAT), on the other hand, comprises mitochondria-rich multilocular adipocytes that specialize in energy dissipation through non-shivering thermogenesis (Chouchani et al., 2019). This evolving view led to a surge of interest in its role in mediating the pathogenesis of CMS (Bjørndal et al., 2011).

### Adipose Tissue Hypertrophy vs. Hyperplasia

Upon an increased caloric intake, exemplified by the consumption of a high-fat diet (HFD) in experimental settings, hypertrophic and hyperplastic AT expansion ensue. As the terms imply, the hypertrophic pathway involves an increase in the size of adipocytes (ACs), while hyperplastic expansion is the increase in the number of ACs. In both settings, these pathways ensures the adaptation of the AT to the positive energy balance, and hence an increased storage capacity of the AT (Choe et al., 2016).

Diet-induced hypertrophy is considered a failure of ACs normal proliferation (Bays, 2011), resulting in malfunctioning ACs that are typically accompanied with cardio-metabolic derangements (Berg and Scherer, 2005; Bays et al., 2008), like hyperinsulinemia, hyperlipidemia, hypertension and atherosclerosis (Gustafson and Smith, 2015). For example, subjects diagnosed with type 2 DM or dyslipidemia were found to have larger subcutaneous ACs than their control counterparts (Berg and Scherer, 2005). The failure of recruitment and differentiation of fat progenitor cells in obesity and prediabetes occurs due to a combination of factors including AT insulin resistance, which provokes the expansion of existing ACs (Gustafson and Smith, 2015). As insulin inhibits lipolysis, insulin resistance leads to increased circulating free fatty acids, which in turn fuels and exaggerates the insulin resistance tightly correlated with hypertension and CVDs at the core of CMS (Bays et al., 2008). In this context, nutrient excess triggers an increased demand for protein and lipid synthesis leading to endoplasmic reticulum stress, which in turn activates Jun kinase and nuclear factor-kB (NF-κB). These latter pathways impair the action of insulin by promoting the inhibitory phosphorylation of insulin receptor substrate 1 (IRS-1). Moreover, it could be plausible that compensatory hyperinsulinemia in obesity or HFD intake may augment AC glucose uptake eventually leading to abnormally hypertrophied ACs in a self-reinforcing vicious cycle (Bays et al., 2008). The vital role of insulin in white AC hypertrophy was confirmed in several animal models. A mouse model of AT-specific insulin receptor knockout showed a significant reduction in total fat mass, age related obesity, and other metabolic abnormalities (Bays, 2011). Moreover, an AT-specific insulin receptor knockout rat model was found to be resistant to diet-induced hypertrophy compared to their wild type littermates (Friesen et al., 2016).

Furthermore, animal studies showed that diet-induced hypertrophy in PVAT was associated with atherosclerosis (Horimatsu et al., 2018), and was correlated to increased vascular tone in aorta and cerebral arterioles (Elkhatib et al., 2019; Fakih et al., 2020). As will be discussed in more detail below, AC enlargement is often associated with reduced oxygen supply subsequently leading to elevated level of hypoxia inducible factor 1-α (HIF1-α), a transcriptional factor that is activated under hypoxic conditions, which promotes the production pro-inflammatory mediators, such as tumor necrosis factor α (TNFα) (van Dam et al., 2017; Agabiti-Rosei et al., 2018). Significantly, incubation of blood vessels obtained from healthy individuals with TNFα abolished the anticontractile effect of PVAT, while PVAT with hypertrophied ACs isolated from obese patients showed no dilator effect (Greenstein et al., 2009). Moreover, as observed in animal studies, hypertrophy is linked to increased production of other pro-inflammatory cytokines, such as Interleukins (IL)-6, IL-8, IL-1β and monocyte-chemoattractant protein 1 (MCP1) (Jernås et al., 2006).

On the other hand, hyperplastic AT expansion, also referred to as adipogenesis (Choe et al., 2016), requires the differentiation of adipose progenitor cells, which are pooled in the AT within the SVF, especially in the white AT (WAT) (Raajendiran et al., 2019). While some adipocyte progenitor cells were identified in murine models (CD31^−^, CD45^−^, CD29^+^, CD34^+^, CD24^+^ and Sca-1+) (Rodeheffer et al., 2008) and in humans (CD44, CD73, CD29 among others) (Raajendiran et al., 2019), the mechanism of *de novo* adipogenesis remains uncertain (Raajendiran et al., 2019). One of the well-established transcriptional factors involved in adipogenesis is peroxisome proliferator-activated receptor-γ (PPARγ), which plays a crucial role in cellular metabolism and the regulation of fatty acids homeostasis (Rosen et al., 1999; Kershaw et al., 2007; Choe et al., 2016). Indeed, the activation of PPARγ2, which is mainly expressed in the AT, promotes adipogenesis by stimulating the differentiation of AC progenitor cells into mature ACs (Tontonoz et al., 1994; Zhang et al., 2004). Importantly, PPARγ expression was found to be higher in the visceral and mesenteric AT of obese diabetic individuals (Yang et al., 2008). Remarkably, obese rats exhibit a lower adipogenic activity in subcutaneous than visceral AT depots including perirenal and gonadal AT (Jamdar, 1978). Intriguingly, an AT-specific PPARγ knockout mouse model was found to be resistant to DIO and subsequent metabolic derangements. Unlike their genetic controls, these mice showed a total lack of brown AT (BAT) in addition to WAT abnormalities including increased fibrosis and vascularization. Yet, they demonstrated normal blood glucose levels, serum triglyceride levels, insulin sensitivity and reduced serum adipokines levels while freely feeding on a high-fat diet (Jones et al., 2005). On another note, deletion of PPARγ in vascular smooth muscle cells in HFD-fed mice, showed its various roles in different AT depots, as it lead to immature mesenteric and perirenal AT, while gonadal, sub-cutaneous, interscapular WAT, and BAT were fully mature (Chang et al., 2012).

### Adipose Tissue Browning or Beiging

Browning or beiging of AT is a process that involves the increase of uncoupling Protein 1 (UCP1) expression. UCP1 is a thermogenic inner mitochondrial membrane protein that uncouples the activity of the electron transport chain from oxidative phosphorylation through the production of a proton leak, and thus dissipates energy in the form of heat in the AT (Ricquier and Bouillaud, 2000; Cannon and Nedergaard, 2017). UCP1 is abundantly expressed in BAT, which specializes in thermogenesis rather than energy storage (Foster and Frydman, 1978; Nedergaard et al., 2001; Kajimura et al., 2015). The regulation of UCP1 expression mainly occurs downstream of *ß*-adrenergic receptors (AR) signaling, more specifically *β*_3-_AR (Cannon and Nedergaard, 2017). Moreover the activation of *β*_3_-AR induces lipolysis, which increases the levels of free fatty acids further enhancing the activity of UCP1 (Coman et al., 2009).

Browning usually occurs in WAT, in order to increase energy expenditure (Nedergaard and Cannon, 2014). Specifically, a recent study showed that AC browning and increased expression of UCP1, among other BAT markers, were observed in several WAT depots in rats receiving a HFD (García-Ruiz et al., 2015). However, murine studies reported an increase in UCP1 expression in BAT as well, in response to HFD feeding (Bonet et al., 2017). Interestingly and from a different perspective, studies showed that inflammation, expected to occur in AT in association with increased caloric intake, downregulates UCP1 expression in WAT and hence, precludes browning (Bartelt and Heeren, 2014). When Adipocytes were challenged with macrophages activated by lipopolysaccharide (LPS) and TNF-α, UCP1 mRNA expression, UCP1 promoter and transcriptional factor binding to cAMP response element were suppressed in WAT in a process mediated by Erk kinase (Sakamoto et al., 2013). Similarly, other studies suggested that UCP1 is downregulated in BAT by low-grade inflammation as seen in mice chronically treated with LPS (Nøhr et al., 2017). In this study, it was proposed that BAT is immunologically naïve since *in vitro* LPS treatment was not able to induce inflammation and cause UCP1 downregulation, which was achieved by direct stimulation with IL-1β instead. A possible pathway for this IL-1β-evoked UCP1 downregulation was proposed to occur through the inhibition of Sirtuin-1 (SIRT1), which stimulates UCP1 expression (Nøhr et al., 2017). This is suggested to occur through upregulating the expression of the endogenous protein deleted in breast cancer-1, an inhibitor of SIRT1. In this regard, SIRT1 plays a crucial role in the AT as it suppresses PPARγ activity in ACs thus decreasing fat accumulation (Rahman and Islam, 2011). This is associated with an increase in lipolysis by inducing mitochondrial fatty acid oxidation, through the activation of the transcriptional factor PPARγ coactivator-1α (PGC-1α) (Gerhart-Hines et al., 1913). In a SIRT1 AC-specific knockout model, normal chow-fed mice exhibited low-grade AT inflammation, along with IR and glucose intolerance in comparison to their wild type littermates (Mayoral et al., 2015). Moreover, this phenotype was exacerbated by short-term HFD consumption. Intriguingly, wild type mice on chronic HFD developed a more pronounced metabolic dysfunction in comparison to their SIRT1 knockout counterparts (Mayoral et al., 2015). Indeed, this was associated with a hyperacetylated PPARγ in the ACs of SIRT1 knockout mice, which correlated with increased dephosphorylation of PPARγ, promoting its constitutive activity that enhances insulin sensitivity (Mayoral et al., 2015). Significantly, a lack of increase in UCP1-mediated energy dissipation in response to HFD consumption triggers BAT changes reminiscent of AT hypertrophy typically seen in WAT depots. Indeed, UCP1 knockout mice receiving HFD exhibited BAT whitening, which was accompanied by an increased AC size and macrophage infiltration (Winn et al., 2017). Additionally, reduced mitochondrial biogenesis, increased endoplasmic reticulum stress, and disrupted glucose tolerance were more pronounced in these mice upon HFD consumption. However, these mice did not show any alterations in visceral adiposity, body weight, energy intake or energy expenditure (Winn et al., 2017). Another important factor is the sympathetic overactivation that is usually triggered by cold exposure. Sympathetic stimulation augments PPARγ-mediated BAT recruitment and proliferation, which in turn was associated with increased expression of UCP1 and mitochondrial biogenesis. This was shown in an *in vivo* study comparing innervated and denervated BAT and demonstrating that PPARγ-mediated UCP1 activation was dependent on the sympathetic nervous system (Festuccia et al., 2010). This is in contrast to *in vitro* research findings, where treating BAT cells with Rosiglitazone, a selective PPARγ agonist, was enough to increase UCP1 and mitochondrial biogenesis in manner that does not involve sympathetic activation (Petrovic et al., 2008). Significantly, it has long been recognized that hyperinsulinemia in early metabolic dysfunction in humans and animals triggers increased sympathetic activity (Anderson et al., 1991; Thorp and Schlaich, 2015).

On the other hand, the increased oxygen consumption associated with browning of white ACs differentiated from human adipose-derived stem cells was correlated with increased mitochondria fission indicated by an increased Dynamin-Related Protein-1 (DRP-1) phosphorylation on serine 616 (Pisani et al., 2018). Moreover, other triggers leading to sub-cutaneous white AC browning were also associated with increased Erk-mediated ser616-DRP1 phosphorylation and mitochondrial fission (Velazquez-Villegas et al., 2018). In response to sympathetic stimulation, BAT adipocytes exhibit a protein kinase A-evoked DRP1 phosphorylation at the same site, enhancing DRP1-mediated mitochondrial fission in order to increase energy dissipation (Wikstrom et al., 2014). The exact molecular relationship between increased UCP1 expression and mitochondrial fission remains unclear. However, increased mitochondrial fission observed under these circumstances has recently been proposed to act as a feedback mechanism increasing the metabolic resilience and protecting against the deleterious effects of increased caloric intake (Rui, 2017). Yet, in the above situations, mitochondrial changes were seen under circumstances not perceived to contribute to AT pathologies. Interestingly, WAT inflammation in diabetic mice was associated with increased ser616-DRP1 phosphorylation. Both observations were reversed when animals received treatments increasing 5′AMP-Activated Protein kinase (AMPK) activity. However, this study did not examine the status of UCP1 expression (Li et al., 2016).

In addition to UCP1, creatine futile cycling is considered as an alternative thermogenic pathway in UCP1-expressing and UCP1-nonexpressing beige ACs (Bertholet et al., 2017). This cycle enhances energy expenditure in beige adipocytes when ADP is limiting, and thus results in a significantly higher oxygen consumption rate through the stimulation of ADP-limited cellular respiration (Kazak et al., 2015). The enzyme creatine kinase (CK) catalyzes the phosphorylation of creatine. Four different CK isoforms have been identified; two cytosolic isoforms, muscle-type creatine kinase (CKM) and brain-type creatine kinase (CKB), and two mitochondrial isoforms, CKMT1 and CKMT2 (Desjardins and Steinberg, 2018). Phosphatases then hydrolyze phosphocreatine into creatine and ATP. Indeed, creatine kinases and creatine phosphatases are compartmentalized within cells to sites of energy production and energy utilization. This uneven distribution of enzymes allows for intercompartmental energy buffering (Wallimann et al., 1992). AC cellular creatine content is either endogenously biosynthesized or imported from the extracellular medium via the creatine transporter Slc6a8 (Kazak and Cohen, 2020). The influx of creatine through Slc6a8 is reduced in states of low energy charge in an AMPK-dependent manner (Li et al., 2010). Also, Slc6a8 expression in human subcutaneous adipocytes is negatively correlated with insulin resistance and BMI (Kazak et al., 2019). In a mouse knockout model of glycine amidinotransferase, the rate limiting enzyme in creatine biogenesis, mice were prone to DIO, had a significantly lower energy expenditure level and exhibited hyperinsulinemia. These metabolic dysfunctions were reversed following creatine administration (Kazak et al., 2017). On another note, intracellular creatine content was depleted following Slc6a8 AC-specific knockout (AdCrTKO). AdCrTKO mice displayed a metabolic response to HFD similar to that shown by glycine amidinotransferase knockouts including reduced energy expenditure and an enhanced weight gain further elucidating the important role of creatine in driving energy expenditure to combat the metabolic challenge (Kazak et al., 2019). Moreover, this type of thermogenesis co-exists with UCP1-dependent thermogenesis in UCP1-positive beige ACs, while it seems to be the only known thermogenic mechanism employed by UCP1-negative beige ones. In comparison to classical UCP1-mediated thermogenesis, creatine cycling seems not to be activated by an acute *β*_3_-AR stimulation but it rather contributes to the modulation of the basal metabolic activity of adipocytes (Bertholet et al., 2017). Importantly, a recent study provided no evidence for creatine supplementation-mediated activation of BAT thermogenesis in acute cold-exposed young, healthy, lean, vegetarian adults, who are characterized by low creatine levels (Connell et al., 2021). This supports previous murine observations where the mere supplementation of creatine is not enough to show the physiological relevance of creatine futile cycling in thermogenesis, if it was not coupled with HFD consumption and *β*_3_-AR stimulation. Moreover, the supplementation of creatine in these individuals does not guarantee an alteration of creatine levels in BAT, and thus, does not necessarily activates the futile cycle.

Another important player in AT remodeling and browning is AMPK (Rui*, 2017). As the deletion of either the *a* or *ß* subunits of AMPK resulted in an impaired WAT beiging with a resistance to *β*_3_-AR stimulation (Mottillo et al., 2016; Wu et al., 2018). In another study, AMPK gain of function mutation induced subcutaneous AT browning. Strikingly, this browning was neither attributed to a detectable increase in UCP1 expression nor to an activated creatine cycling, despite the increase of oxygen consumption in this depot (Pollard et al., 2019). Nevertheless, this does not exclude a role of creatine cycling in enhancing energy expenditure and oxygen consumption in other adipose depots that have not yet been investigated.

### Adipose Tissue Inflammation

As discussed above, increased caloric intake-induced hyperinsulinemia drives AC hypertrophy, promoting its diametric expansion beyond the diffusion potential of oxygen (Pedersen et al., 2015). Importantly, this occurs in the absence of compensatory AT vascularization creating a local hypoxic state that is associated with an increased expression of HIF-1α (Ye et al., 2007; He et al., 2011; Trayhurn, 2013). An extensive crosstalk between signaling pathways involving HIF-1α and other transcription factors implicated in the AT hypoxic response such as NF-κB occurs, where NF-κB was shown to regulate HIF-1α transcription (Rius et al., 2008; van Uden et al., 2008; Trayhurn, 2013). Additionally, it was shown that hypoxia-triggered expression of HIF-1α induces NF-κB-mediated cytokine production including IL-1β, which results in subsequent recruitment and accumulation of distinct populations of immune cells giving rise to a state of chronic AT inflammation (Jeong et al., 2005; Fitzgibbons and Czech, 2014; Dzhalilova et al., 2019; Saxton et al., 2019). Importantly, the dysfunction of adipose depots implicated in the pathogenesis of CMS, such as epicardial, perivascular, and perirenal adipose depots, is associated with a perturbed immune cell landscape and adipokine profiles in states of metabolic dysfunction (Alzaim et al., 2020).

Adipose tissue macrophages (ATMs) exhibit remarkable polarization-dependent transcriptomic heterogeneity that is highly dependent on microenvironmental factors (Thapa and Lee, 2019; Caslin et al., 2020). AT-resident or infiltrating macrophages can either adopt a classical, pro-inflammatory M1 polarization, or an anti-inflammatory M2 polarization. ATMs in lean AT are predominantly M2 polarized (Morris et al., 2011; Chylikova et al., 2018), while M1 macrophage infiltration into inflamed ATs as well as the phenotypic switch of resident M2 macrophages to M1 macrophages occur in response to HFD consumption (Lumeng et al., 2008; Kralova Lesna et al., 2016). As such, these macrophages associate with crown-like structures (CLS), which represent macrophages actively phagocytosing apoptotic adipocytes with the concurrent increased production of proinflammatory cytokines and chemokines as well as reactive oxygen species (ROS) (Sartipy and Loskutoff, 2003; Weisberg et al., 2003; Cinti et al., 2005; Lumeng et al., 2007; Wensveen et al., 2015; Chylikova et al., 2018).

Moreover, it was suggested that ATMs represent the primary source of the proinflammatory cytokines TNFα, IL-1, IL-6 and iNOS (Weisberg et al., 2003; Xu et al., 2003). Noteworthy, it was suggested that WAT preadipocytes can undergo a phenotypic switch, by which they transdifferentiate into macrophages *in vivo* in response to HFD under a contact-dependent macrophage-mediated stimulation (Charrière et al., 2003; Xu et al., 2003). Nevertheless, *in vitro* studies suggest that AC-macrophage crosstalk is mainly mediated by free fatty acids (FFA) and TNFα. Indeed, TNFα was shown to drive AT inflammation and to reduce adiponectin expression, while FFAs were found to increase macrophage cytokines production (Suganami et al., 2005). This crosstalk was found to be exaggerated upon the use of hypertrophied or obese ACs (Suganami et al., 2005). Accumulating evidence suggests a role for TNFα in inhibiting PPARγ activity through several pathways (Ye, 2008), among which is the activation of the classical NF-κB pathway, which prevents PPARγ binding to its response element, and hence blocks its downstream effect (Suzawa et al., 2014). As PPARγ represents a major promoter of adipogenesis, it is thought that TNFα-mediated suppression of PPARγ signaling would increase levels of circulating FFAs and subsequently enhancing the proinflammatory polarization of ATMs (Ren et al., 2006). This is supported by evidence from PPARγ receptor agonist treated HFD-fed mice, which exhibited an enhanced overall insulin sensitivity and an increased M2 count in VAT (Fujisaka et al., 2011). Indeed, TNFα KO mice were protected from HFD-induced IR and exhibited reduced serum FFA levels (Uysal et al., 1997). Moreover, ATM-derived TNFα is suggested to be the leading promoter of adipose-specific insulin resistance through various mechanisms (Ruan et al., 2002; Xu et al., 2003; Cawthorn and Sethi, 2008). It was shown that TNFα downregulates the expression of insulin receptor substrate 1 (IRS-1) and glucose transporter type 4 (GLUT4) (Stephens et al., 1997; Engelman et al., 2000), and inhibits the activity of AMPK activity (Steinberg et al., 2006). In addition to AMPK energy sensing activity, and the crucial role of AMPK signaling dysfunction in the pathogenesis of IR, AMPK activation was shown to prime M2 macrophage polarization (Chan et al., 2015).

Although ATMs are considered the main contributors to HFD-induced AT inflammation (Weisberg et al., 2003), other distinct immune cell populations were also found to be involved (Alzaim et al., 2020). For example, dendritic cells were shown to regulate AT inflammation, where the accumulation of dendritic cells in the AT of HFD-fed mice promotes the recruitment of macrophages and the mounting of a Th17-driven inflammatory response (Bertola et al., 2012; Stefanovic-Racic et al., 2012; Chen et al., 2014). Moreover, the uptake of FFAs by dendritic cells and the formation of lipid droplets is suggested to promote dendritic cell immunogenicity, which likely occurs in states of metabolic dysfunction (den Brok et al., 2018). Moreover, neutrophils, which are relatively rare in the AT of lean mice, exhibit a maintained flux into the inflamed AT of HFD-fed mice, whereby they promote IR and the production of proinflammatory cytokines (Talukdar et al., 2012). Neutrophils accumulation in inflamed AT is thought to precede and subsequently enhance macrophage infiltration through the increased activity of NF-κB and production of IL-1β (Watanabe et al., 2019). Similarly, mast cells are enriched and further increase in VAT of mice and humans in states of obesity and T2D, where they drive macrophage infiltration and AT inflammation (Nishimura et al., 2009b; Liu et al., 2009; Żelechowska et al., 2018; Kumar et al., 2019). It was also suggested that mast cell accumulation in inflamed AT occurs prior to overt obesity and the genetic ablation or functional impairment of mast cells in HFD-fed mice decreased weight gain and reduced IR (Nishimura et al., 2009b; Liu et al., 2009). Alternatively, AT-resident eosinophils promote AT homeostasis through the secretion of IL-4 and IL-13 which promotes M2 macrophage polarization, triggers Th2 differentiation, and enhances B cell activation (Wu et al., 2011; Yoon et al., 2019). The homeostatic role of eosinophils is supported by the observation that eosinophil-deficient HFD-fed mice show pronounced IR while increasing eosinophil abundance in the AT reduced HFD-induced increased adiposity (Wu et al., 2011; Molofsky et al., 2013; Lee et al., 2018). Moreover, the forced increase of AT eosinophil abundance in different models enhanced metabolic homeostasis (Wu et al., 2011; Qiu et al., 2014; Berbudi et al., 2016).

T and B lymphocytes also play major immunoregulatory roles in AT homeostasis and dysfunction. Different subsets of proinflammatory T cells increase in visceral adipose depots of mice and humans including helper T cells, γδ T cells, and cytotoxic T cells and drive IR (Weinstock et al., 2020). Conversely, the abundance of anti-inflammatory T cells such as regulatory T cells and invariant killer T cells, decreases in obesity (Feuerer et al., 2009; Deiuliis et al., 2011). It was proposed that T cell infiltration into inflamed AT precedes that of macrophages with particular enrichment of CD4^+^ T cells (Kintscher et al., 2008; Shen et al., 2015). Also, expanding regulatory T cells in HFD-fed mice was shown to alleviate HFD-induced metabolic dysfunction (Ilan et al., 2010). B cells accumulation in AT also modulates AT inflammation. For example, regulatory B cells suppress Th1 and Th2 polarization, as well as inhibit macrophage and dendritic cell activation (Alzaim et al., 2020). Moreover, different subpopulations of B cells such as B-2 cells are thought to promote AT inflammation (Winer et al., 2011; Defuria et al., 2013; Ying et al., 2017), while B-1 cells improve glucose tolerance and reduces AT inflammation through the induction of M2 macrophage polarization and IL-10 secretion with simultaneous reduction in the production of IL-6 and TNF-α (Wong et al., 2010; Wu et al., 2014; Shen et al., 2015; Harmon et al., 2016; Srikakulapu et al., 2017).

Of particular relevance to the pathogenesis of the CMS, PVAT was recently shown to harbor a plethora of basally activated immune cells under homeostatic conditions including CD4^+^ and CD8^+^ T cells, natural killer cells, B cells, macrophages, mast cells, and neutrophils (Kumar et al., 2020). Metabolically dysfunctional PVAT is infiltrated by macrophages, T cells, NK cells, and DCs that produce either pro-inflammatory or anti-inflammatory cytokines depending on PVAT adipokine profile shifts (Saxton et al., 2019). As such, accumulating evidence implicates local alterations of resident and infiltrating immune cell populations within the SVF in AT inflammation and the pathogenesis of insulin resistance, metabolic syndrome, and diabetes (Alzaim et al., 2020). Moreover, AT dysfunction is associated with an imbalanced adipokine profile that further promotes detrimental AT immune cell landscape shifts (Alzaim et al., 2020).

## Adipose Tissue and Cardiometabolic Syndrome

AT affect cardiac health through paracrine and endocrine interactions with cardiovascular tissues making it prudent to study AT inflammation in the context of CMS (Berg and Scherer, 2005; Shah et al., 2008). Significantly, excessive calorie intake, DIO and T2DM are correlated with a wide range of CVDs while sharing a background of low-grade AT inflammation (Nishimura et al., 2009a; Klöting and Blüher, 2014). One example is the increase of proinflammatory macrophages, cytokines, and reactive oxygen species in epicardial AT in patients with coronary artery diseases (Eiras et al., 2008; Salgado-Somoza et al., 2010; Hirata et al., 2011). Another important one is cardiac autonomic neuropathy (CAN) showing as reduced parasympathetic activity and endothelial dysfunction, both major risk factors predisposing to CVD associated with metabolic impairment (Bakkar et al., 2020a). CAN is known to occur early in the course of metabolic derangement exemplified by the prediabetic stage (Gonzalez and Selwyn, 2003; Vinik et al., 2013; Williams et al., 2019). In fact, obese patients with insulin resistance had systemic arterial dysfunction concurrent with high M1 ATMs and TNFα mRNA in SAT and elevated serum C reactive protein (CRP), indicating a proinflammatory state (Apovian et al., 2008). Interestingly, recent studies on a prediabetic rat model showed that mildly increased caloric intake led to clear cardiovascular manifestations even in absence of overt hyperglycemia, increased body weight, or high blood pressure (Al-Assi et al., 2018; Alaaeddine et al., 2019; Elkhatib et al., 2019). Significantly, the observed endothelial dysfunction and CAN were linked to localized PVAT inflammation with neither inflammatory changes in other adipose pool nor systemic involvement (Al-Assi et al., 2018; Alaaeddine et al., 2019; Elkhatib et al., 2019). This early PVAT involvement in the course of metabolic disease is of particular relevance given the close anatomical proximity between PVAT and the cardiovascular tissue. While the role of PVAT has been initially limited to vascular supportive and structural functions, its ability to modulate vascular contractility has long been recognized (Verlohren et al., 2004; Galvez et al., 2006; Gao et al., 2006; Gao et al., 2007; Greenstein et al., 2009). Mechanistic pathways by which PVAT secretes a complex array of factors to modulate vascular tone have been proposed. Indeed, as early as 1991, periaortic fat was shown to exert an anti-contractile effect (Soltis and Cassis, 1991) that persisted when vessels without AT were treated with PVAT-conditioned media (Lohn et al., 2002). In this regard, PVAT adipokines are highly likely to exert direct effects on the nearby vascular tissue. Of these, adiponectin was shown to mediate the anticontractile effect via eNOS stimulation (Sung et al., 2004; Ouchi et al., 2011). Additionally, animal models of deleted PPARγ that lacked the PVAT depot, showed endothelial dysfunction and increased cardiovascular diseases (Chang et al., 2012) supporting the fundamental role of PVAT in modulating cardiovascular health (Agabiti-Rosei et al., 2018; Qi et al., 2018). Of note, studies have shown that the anticontractile effect is lost in metabolic diseases like diabetes, as PVAT phenotype shifts into a proinflammatory state (Azul et al., 2019). This is accompanied by significant perturbation in the adipokine profile involving several of these products including adiponectin, leptin, chemerin, resistin, and visfatin as described previously in detail (Rafeh et al., 2020).

**FIGURE 1:**
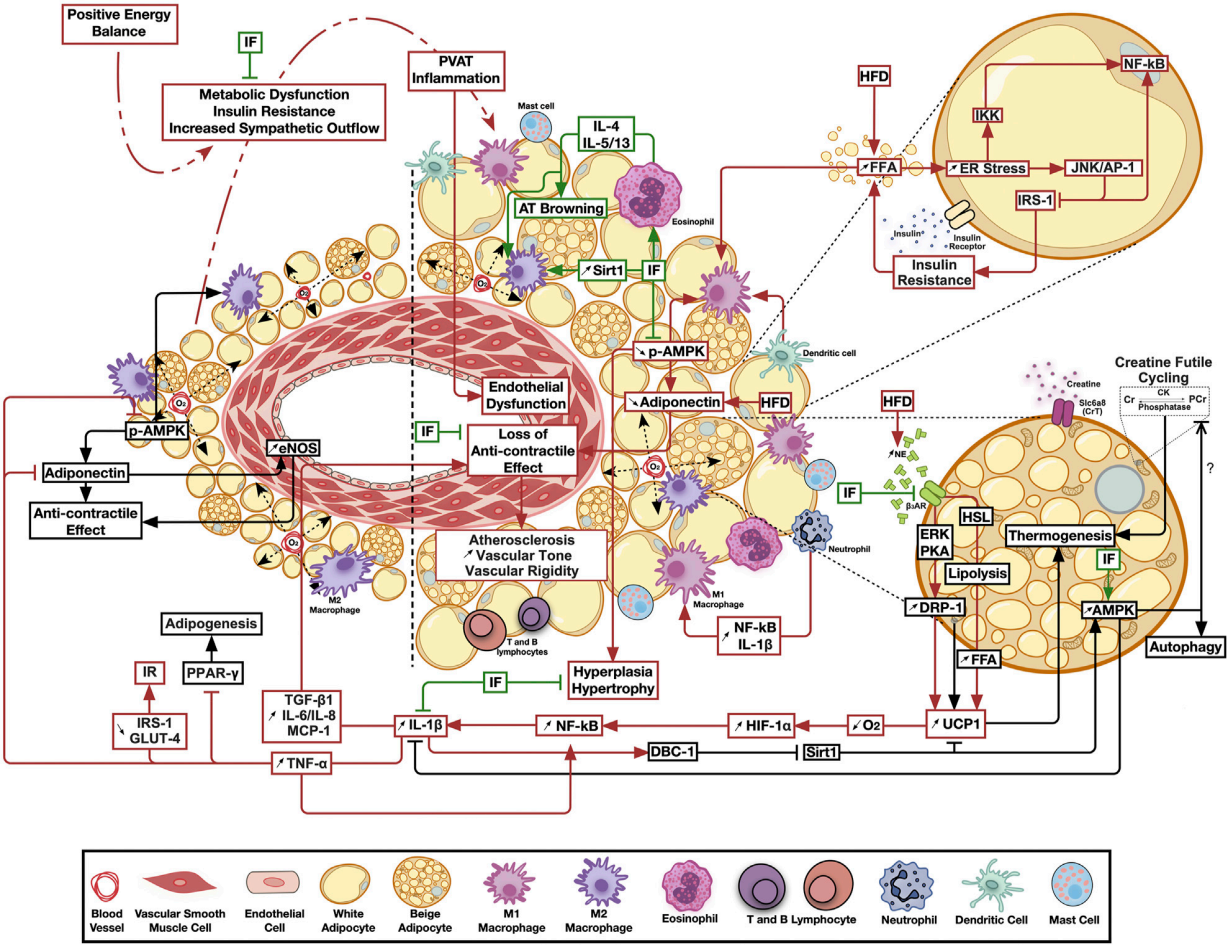
The emerging ameliorative role of intermittent fasting on perivascular adipose tissue inflammation and thermogenesis. Calorie excess resulting in metabolic dysfunction triggers perivascular adipose tissue chronic low-grade inflammation, leading to the loss of its anticontractile effects and the subsequent negative paracrine modulation of vascular structure and function. Intermittent fasting ameliorates inflammatory, thermogenic and bioenergetic pathways favoring adipose tissue homeostasis. Pathways involved in adipose tissue homeostasis are shown in black, those activated by metabolic dysfunction in red, while those modulated by intermittent fasting in green arrows. AT, Adipose tissue; AMPK, AMP-activated protein kinase; β_3_AR, Beta 3 adrenergic receptor; CK, Creatine kinase; Cr, Creatine; DBC-1, Deleted in bladder cancer protein 1; DRP-1, Dynamin related protein 1; eNOS, Endothelial nitric oxide synthase; ER, Endoplasmic reticulum; ERK, Extracellular signal-regulated kinase; FFA, Free fatty acids; HFD, High fat diet; HIF-1α, Hypoxia-inducible factor 1-alpha; HSL, Hormone sensitive lipase; IF, Intermittent fasting; IKK, IKβ kinase; IL, Interleukin; IRS-1, insulin receptor substrate 1; JNK/AP-1, c-jun N-terminal kinase/activator protein 1; MCP-1, Monocyte Chemoattractant protein 1; NE, Norepinephrine; NF-κB, Nuclear factor kappa B; O_2_, Oxygen; PVAT, Perivascular adipose tissue; PCr, Phosphocreatine; PKA, Protein kinase A; Sirt1, Sirtuin 1; TGF-β1, Transforming growth factor beta 1; TNF-α, Tumor necrosis factor alpha; UCP1, Uncoupling protein 1.

Noteworthy, PVAT in postmenopausal women was found to have a higher number of proinflammatory macrophages, compared to other depots, and it was suggested that this is associated with increased CV risk (Kralova Lesna et al., 2016). Another study indicated that upon the transplant of thoracic aortic PVAT from HFD fed mice to the carotid artery of HFD-fed ApoE^−/-^ mice vascular injury was augmented, and it was mediated by MCP1 expression (Manka et al., 2014). It also was suggested that proinflammatory ATMs migration induces inflammation in the vascular bed and poses atherogenic effect (Britton and Fox, 2011). Furthermore, it appears that PVAT is especially sensitive to hypoxia-driven inflammation (Greenstein et al., 2009). Indeed, isolated PVAT inflammation, with significant implications on vascular structure and function, was observed in more than one animal model of metabolic challenge. Transplantation of inflamed PVAT from HFD-fed ApoE^−/−^ mice increased the incidence of atherosclerosis and endothelial dysfunction in recipient animals on a control diet (Horimatsu et al., 2018). Moreover, prediabetic rats fed mild hypercaloric diet exhibited an increased expression of PVAT UCP1, DRP1 as well as HIF1-α, in addition to a hypertrophied inflamed morphology that were associated with vascular dysfunction in absence of similar changes in other adipose depots (Elkhatib et al., 2019). As such, one would assume that this peculiar nature, whereby several PVAT pools expressed brown adipose-specific genes (Chatterjee et al., 2009; Sacks et al., 2013; Gaborit et al., 2015), might be the cause of the observed early involvement and increased sensitivity to inflammation. In this context, an assumed exaggerated oxygen consumption triggered by increased UCP1 expression would be exacerbated by the observed adipocyte hypertrophy in a combination of events less likely to occur in other adipose depots. This model is illustrated in Figure 1. Importantly, another rat model on HFD showed increased UCP1 expression in PVAT, however this increase was not explained nor were its implications investigated (Winn et al., 2017). On the other hand, HFD reduced UCP1 in WAT and VAT, supporting the hypothesis indicating that AT depots act varyingly in response to the same energy stimuli (Winn et al., 2017). As for creatine cycling, it remains widely unknown if the creatine-mediated enhanced basal metabolic activity may predispose PVAT to hypoxia following HFD consumption, or if this enhancement will limit cellular thermogenic needs, and thus limit UCP1 induction. Indeed, it has been suggested that creatine cycling and UCP1-dependent thermogenesis represent parallel thermogenic pathways that are independently operational and potentially reciprocal (Kazak and Cohen, 2020). Of relevance to inflammatory changes, Slc6a8-mediated creatine uptake and accumulation stimulate macrophages reprograming by inhibiting the pro-inflammatory M1 and promoting the tissue repair-responsible M2 polarization in peritoneal macrophages via regulating cytokine responses (Ji et al., 2019). Moreover, accumulating evidence suggests the implication of creatine metabolism in mediating anti-inflammatory phenotypes in immune cells (Kazak and Cohen, 2020).

In the above rat model of early metabolic dysfunction, localized PVAT inflammation led to increased IL-1β and TGF-β1 production, which was associated with reduced AMPK activation, increased vascular Erk1/2 phosphorylation, medial hypertrophy, oxidative stress, increased rho-associated kinase (ROCK)-mediated calcium sensitization and a hypercontractile response (Elkhatib et al., 2019; Lefranc et al., 2019). This isolated PVAT inflammation model was also associated with impaired endothelial relaxing function due to reduced expression/function of inward rectifier K^+^ channels (Alaaeddine et al., 2019). Treatment of the local PVAT inflammation ameliorated the contractile and endothelial manifestations. A different animal study using *AMPKα1* knockout mice assessed the impact of HFD on PVAT function. HFD in wild-type mice reduced P-AMPK and adiponectin levels, in addition to diminished anti-contractile involvement of PVAT accompanied by an infiltration of macrophage with M1 polarization, indicated by increase in iNOS and IL-1 *ß*. Yet in *AMPKα1* KO mice, PVAT of both HFD and control fed animals showed a massive reduction in P-AMPK, adiponectin and abolished vasorelaxation (Almabrouk et al., 2018). More recent investigation showed that not only is the occurrence of PVAT inflammation linked to the early incidence of cardiovascular manifestations associated with metabolic dysfunction, but is also necessary for the progression of cardiac autonomic insult as the metabolism deteriorates further ending up in overt hyperglycemia and diabetes (Bakkar et al., 2020b). Taken together, one can conclude that HFD-induced negative remodeling of PVAT lead to a state of cardiometabolic dysfunction.

## Adipose Tissue Remodeling in Response to Therapeutic or Intermittent Fasting

Intermittent Fasting was found to produce a positive impact on AT remodeling occurring in diet-induced metabolic dysfunction. After eight weeks of treatment, not only did IF (24-h fast on three nonconsecutive days per week) improve glucose tolerance, and insulin resistance in HFD-fed mice, it also reduced adipocyte hypertrophy and markers of inflammation including macrophage infiltration and the NLRP3 inflammasome signaling components (Liu et al., 2018). Moreover, the same IF protocol as well as a complete alternate-day fasting protocol evoked an increased energy expenditure and UCP1 expression in WAT tissue in DIO mice in a manner that also involved reduction of inflammatory markers (Liu et al., 2019b; de Souza Marinho et al., 2020). Yet, while IF reduced WAT fat mass in both obese mice models and obese human subjects, no UCP1 upregulation was observed in humans (Liu et al., 2019b). Nevertheless, the exact mechanism of how these fasting regimens improve the metabolic state are still not fully understood. Specifically, despite the many desired outcomes reported for calorie restriction, isocaloric IF seems to have a prominent effect on the metabolic health, despite the fact in a lot of studies did not involve calorie restriction (Aksungar et al., 2017; Kim et al., 2017; St-Onge et al., 2017).

In murine models, HFD-induced hypertrophy was reported to be reversed by 24-h and 72-h IF in VAT; especially gonadal and subcutaneous inguinal AT depots (Tang et al., 2017). Interestingly, the change in fat pad size was found to be triggered by IF alone, regardless of the daily caloric intake, and independent of the change in body weight as animal studies showed a decrease in size and weight of adipose depots, with mild or no change in total body weight (Varady et al., 2007; Varady et al., 2009; Varady et al., 2010; Liu et al., 2019b). Certainly in our work, a calorie restricted regimen of a HFD failed to exert any corrective effect on CAN and PVAT inflammation involvement in rats (Al-Assi et al., 2018) as opposed to isocaloric IF (12-h feeding/12-h fasting) (Dwaib et al., 2020). In studies involving obese human subjects, IF appeared to reduce total fat mass with varying fasting protocols such as intermittent continuous energy restriction for 2 days a week and alternate day fasting (Harvie et al., 2011; Bhutani et al., 2013; Tinsley and La Bounty, 2015), and circulating markers of inflammation (Harvie et al., 2011; Wang et al., 2020), the latter effect being more marked in obese than in normal weight subjects (Wang et al., 2020).

In further confirmation of the observations in animal studies, IF regimens were better capable of reducing circulating markers of inflammation than calorie restricted regimens *per se* (Wang et al., 2020). Nonetheless, mixed results were found in human studies. In a study examining IF during the month of Ramadan, which is the religious fasting in Islam involving fasting from sunrise till sunset for 30 days, body weight and fat percentage decreased in both men and women in absence of calorie restriction (Faris et al., 2012). Meanwhile, others reported that the IF-induced weight loss was dependent on calorie restriction that mediated the metabolic benefit rather than the fasting regimen itself (Klempel et al., 2012; Patterson and Sears, 2017). Moreover, a recent clinical trial comparing the effects of calorie restriction to IF (24-h fast on three nonconsecutive days per week) showed a transient increase in inflammatory markers in AT after IF only, which was attributed to an ATM response to increased lipolysis (Liu et al., 2019a). Still in either case, a marked overall improvement in AT functioning was observed.

As such, IF might constitute an adequate intervention in the low-grade inflammatory state evoked in AT by DIO. Indeed, IF produced the previously mentioned anti-inflammatory effects in metabolically challenged mice alongside reduction of body weight and insulin resistance (Liu et al., 2018). Interestingly, isocaloric IF was shown to produce the same effects and trigger alternative activation of macrophages to the M2 polarization in mice (Kim et al., 2017). Another murine study involving IF in a calorie restriction protocol showed a shift in AT macrophages to the M2 polarization that was mediated by SIRT1 activity (Fabbiano et al., 2016). SIRT1 is a nutrient sensitive histone deacetylase that is thought to mediate the beneficial metabolic effects of fasting and calorie restriction including improved serum glucose and lipid levels, increased insulin sensitivity and reduced body weight (Bordone et al., 2007; Schenk et al., 2011). Specifically, mice mildly overexpressing SIRT1 were protected against HFD-induced inflammation through a reduction of NF-κB activation and pro-inflammatory cytokine production (Pfluger et al., 2008). Pertinent to the inflammatory context in AT, SIRT1 activation was shown to mitigate hypoxic cardiomyocyte damage through the augmentation of autophagic flux that was mediated by AMPK activation (Luo et al., 2019). Indeed, IF (15 or 39 h fast) was found to augment AMPK phosphorylation in rats (Kajita et al., 2008). Significantly, not only has our work shown a reduced AMPK activity in cardiovascular impairment associated with early metabolic dysfunction involving PVAT inflammation in HFD-fed rats (Al-Assi et al., 2018), our results also demonstrated autophagy suppression as a possible contributor to the observed phenotype (Bakkar et al., 2020b; Fakih et al., 2020). Moreover, a lack of AMPK activity was implicated in AC hypertrophy (Villena et al., 2004). As such, IF-mediated SIRT1 activation could offer a mechanistic link for the observed positive changes in AT remodeling. However, it is important to note that studies on human subjects reported inconsistent results where some studies showed increased SIRT1 expression in AT following 6 days of total fasting (Pedersen et al., 2008), and others have reported no change (Madkour et al., 2019). These observations could be related to a lack of consistency in the fasting regimens employed and warrant further investigation.

On the other hand, murine studies employing different IF regimens have been shown to strongly induce WAT browning and increase the expression of mitochondrial UCP1, which was linked to an improved metabolic state (Fabbiano et al., 2016; Kim et al., 2017; Kivelä and Alitalo, 2017; Liu et al., 2019b). Indeed, this could be related to SIRT1 activation that was shown to increase UCP1 expression as mentioned previously (Nøhr et al., 2017). Yet, data from mouse models, showed that caloric restriction induces browning in SAT and VAT, via eosinophils infiltration, M2 macrophage polarization and anti-inflammatory cytokines production. Genetic ablation of the effect of these cytokines (IL-4,-5 and -13) abolished AT browning along with the observed improvement of AT inflammation and metabolic parameters (Fabbiano et al., 2016). Significantly, another aspect of mitochondrial function involves AMPK activity. Evidence suggests that fasting-mediated AMPK activity has a role in mitochondrial metabolism and homeostasis, where chronic activation of AMPK maintains the dynamic nature of mitochondrial networks together with their ability to interact with other organelles and increase fatty acid oxidation (Weir et al., 2017). Furthermore, AMPK phosphorylates and inhibits creatine kinase and thus impedes creatine cycling in skeletal muscle (Ponticos et al., 1998; Weir et al., 2017). Such an effect would reduce basal mitochondrial oxygen consumption and help mitigate AT hypoxia. However, though emerging evidence implicates AMPK in the modulation of AC phenotype plasticity, AMPK-mediated inhibition of creatine kinases in ACs has not yet been investigated (Desjardins and Steinberg, 2018). This further extends the mechanism by which the beneficial metabolic effect of IF may arise via AMPK-activation. Indeed, the increased AMPK activity is consistent with the effect of IF on adiponectin production. A localized increase in adiponectin in AT, especially WAT, was achieved by different IF protocols even in the presence of HFD. These observations were consistent in both human and animal studies (Barnosky et al., 2014a; Patterson et al., 2015; Lettieri-Barbato et al., 2016; Antoni et al., 2017; Kim et al., 2017). However, the effect of IF on adipokine profile alteration as well as its role in immune cell metabolic rewiring subsequent to such metabolic challenges warrant further investigation, especially that specific alterations in adipokine profile were shown to adversely affect immune cell recruitment and polarization.

On another note, fasting was suggested to activate autophagy as cells tend to recycle intracellular particles to produce energy in several tissues including AT (Bagherniya et al., 2018; Ferhat et al., 2019; Clemente-Postigo et al., 2020). IF has been linked to the upregulation of several autophagy-related genes, microtubule-associated protein 1 light chain 3 (LC3), Beclin1, lysosomal associated membrane protein, Unc-51-like kinases (ULK1), in addition to SIRT1 (Bagherniya et al., 2018), which are involved in different parts of the autophagy machinery; initiation, nucleation, autophagosome formation, maturation and cargo degradation (Ferhat et al., 2019; Clemente-Postigo et al., 2020). Significantly, a crossover randomized trial of 18 h daily fasting for 4 days found to improved glucose balance and lipid metabolism alongside with increased serum SIRT1 and LC3A in overweight adults (Jamshed et al., 2019). Indeed, the autophagy machinery is managed by the major nutrient sensing kinases mTORC1 and AMPK, which inhibit and activate autophagy, respectively (Ferhat et al., 2019; Lahiri et al., 2019; Clemente-Postigo et al., 2020). Persistent inhibition of autophagy in response to overnutrition has determinantal effects on the metabolic state (Singh et al., 2009a; Ro et al., 2019). As such, IF-mediated AMPK activation might be a possible pathway through which therapeutic fasting can alleviate metabolic dysfunction in adipose tissue by achieving autophagic homeostasis.

Data on the level of autophagy in adipose tissue have been conflicting. Some studies reported that autophagy is overactivated in obese WAT where autophagy suppression was suggested to inhibit WAT adiposity in DIO (Ro et al., 2019). On the other hand, acute starvation at different time points (12–72 h) after HFD feeding was found to induce lipophagy and lipolysis by increased *ß*-oxidation (Singh et al., 2009a). Another study reported that VAT of obese and insulin resistant indivduals had hypertrophied ACs and increased mRNA expression of autophagy genes compared to subcutaneous WAT (Kovsan et al., 2011). Moreover, autophagy inhibition or more specifically blocking of mitophagy in WAT leads to a state of browning, due to the accumulation of mitochondria (Altshuler-Keylin and Kajimura, 2017). A study on HFD-fed rats found that pharmacological inhibition of autophagy induced WAT browning and protected against DIO (Parray and Yun, 2017). On the contrary, it was suggested that obesity is correlated with increased dysfunctional mitochondria in WAT (Chattopadhyay et al., 2015), which might be a sign of impaired mitophagy in AT in response to DIO (Ro et al., 2019). Atg7 knockout mice demonstrated a lean phenotype with increased BAT, *ß*-oxidation and browner WAT (Singh et al., 2009b). While seemingly conflicting, these findings emphasize the vital role of autophagy in maintaining AT plasticity in terms of thermogenic activity (Cairó and Villarroya, 2020).

Importantly, one study examined the impact of short-term fasting for 24 h on genomic profile of WAT and BAT in male Wister rats. Autophagy gene expression increased in BAT of fasted rats (Nakai et al., 2008). Another mouse model fed an isocaloric fasting regimen, which provides food in 12-h inter-meal cycles, showed increased autophagy, improved body compositions, reduced AC size reduced adiposity and enhanced muscle mass compared to ad libitum fed controls. Interestingly, M2 polarization was observed in ATM (Martinez-Lopez et al., 2017). These changes were found to be autophagy dependent, as they were abolished in AT Atg7 knockout mice regardless of the dietary regimen. Moreover, the knockout mice had impaired glucose tolerance and insulin sensitivity in response to inter-meal fasting compared to the wild-type controls, suggesting the role of AT autophagy in modulating glucose homeostasis.

Nevertheless, all of the above observations were reported in large WAT pools including sub-cutaneous, gonadal, and other visceral AT depots. To the best of our knowledge, there is no direct investigation of the effect of IF on PVAT in situations of metabolic dysfunction. As such, a systematic examination of the impact of IF on PVAT remodeling and inflammation in early metabolic dysfunction and its impact on cardiovascular impairment is warranted. While several parallels among that responses of different WAT pools to IF can be drawn, one must be cautious in extrapolating these findings to PVAT given the peculiar nature of this adipose pool. In this context, prolonged periods of reduced caloric intake, as in case of IF, might, in addition to the previously observed effects in other AT depots, exert further benefit by relieving the UCP1-mediated exacerbation of oxygen deficiency, and hence ameliorate the early inflammatory response. This framework is depicted in Figure 1.

## Cardiometabolic Syndrome and Therapeutic or Intermittent Fasting

The positive impact of IF on AT is expected to ameliorate the cardiovascular manifestations of cardiometabolic syndrome. Indeed, the American heart association (AHA) included IF as one of the dietary measures to prevent CVDs. Based on human studies, AHA concluded that IF, regardless of its effect on weight, improves lipid profile, lowers LDL and cholesterol and increases HDL, in addition to improving insulin sensitivity, indicated by reduced HOMA-IR, with no change in blood glucose level (St-Onge et al., 2017). As well, human and animal studies indicated that alternate day fasting and religious Ramadan fasting IF protocols reduced blood pressure (Wan et al., 2003; Lotfi et al., 2010; Erdem et al., 2018) and heart rate (Lotfi et al., 2010). Another study on a rat model found that both 40% calorie restriction and alternate day fasting reduced the low frequency component in the diastolic blood pressure variability, a marker of reduced sympathetic activity, and increased high frequency component in heart rate variability, which is reflective of the parasympathetic tone, both being indicative of positive modulation of cardiovascular state (Mager et al., 2006). Specifically, in the context of early metabolic dysfunction, preliminary data indicate that not only did IF (12-h feeding/12-h fasting) improve parasympathetic cardiac autonomic neuropathy, this was also associated with amelioration of PVAT inflammation in an HFD-fed rat model of metabolic dysfunction (Dwaib et al., 2020).

Furthermore, IF (19-h fast daily for 26 days) improved endothelial and non-endothelial dependent vasorelaxation in healthy men (Esmaeilzadeh and Van De Borne, 2016). Significantly, alternate day IF exerted a similar pattern in Wistar male rats, as it showed an improved aortic endothelial depend relaxation (Razzak et al., 2011). Moreover, IF is found to prevent atherosclerotic state by promoting an anti-inflammatory response (Malinowski et al., 2019). However, mechanistic data describing the impact of IF on cardiovascular remodeling is scarce. A recent study examined the effect of alternate day fasting on cardiac remodeling post-myocardial infarction (MI) in rats. While IF improved cardiac function and reduced left ventricular dilation, none of the fibrosis gene markers examined appeared to have been affected (Okoshi et al., 2019). Another study showed that alternate day fasting promoted cardiomyocyte survival post-MI in rats by increasing the expression of anti-apoptotic and angiogenic factors (Katare et al., 2009). Interestingly, the post-MI protective effect of alternate day fasting was suggested to be mediated by an increase in adiponectin (Wan et al., 2010) further implicating the normalization of AT homeostasis as a possible mediator of the observed beneficial effect. Noteworthy, prophylactic IF was found to be protective against tissue and neurological damage caused by ischemic stroke. It works mainly on reducing inflammatory cytokines (IL-1β, IL-6, TNFα among others), inflammasome activation in the stroke side of the brain and oxidative stress, while increasing autophagy, mitophagy and neuroprotective proteins like; neurotrophic factors (BDNF and bFGF), antioxidants enzymes (HO-1), UCP-2 and UCP-4 (Fann et al., 2017). In this context, it was also found the fasting mediates its beneficial effects by increasing neuronal and glial SIRT-1 and P-AMPK (Fann et al., 2017).

Literature summarizing the impact of various dietary interventions on cardiovascular disease makes little discrimination between the effect of intermittent fasting and calorie-restrictive diets on cardiovascular outcomes (Maugeri and Vinciguerra, 2020). However, a recent trial directly compared an eight-week protocol of intermittent calorie restriction (3 days/week of low-calorie intake) to chronic reduced calorie intake in humans with mild metabolic impairment (overweight with mild hypertriglyceridemia). The results showed that while both protocols had an equal impact on body weight, body composition, and blood lipid levels, intermittent lowered insulin resistance further possibly having a more profound impact on AT inflammation and cardiovascular risk (Maroofi and Nasrollahzadeh, 2020). Moreover, another clinical trial specifically comparing the post-prandial indices of cardiometabolic risk between intermittent calorie restriction for two days per week and chronic calorie restriction in protocols extending to 3 months found that intermittent restriction was more effective in reducing post-prandial lipemia and insulinemia (Antoni et al., 2018). Nevertheless, a recent systematic review of clinical studies comparing IF to chronic calorie restriction found intermittent fasting to be more effective in weight reduction without a clear effect on blood glucose levels (Allaf et al., 2021). Indeed, this confirms the view of earlier investigation that different fasting protocols were not clearly effective in reducing blood glucose levels, and hence effectiveness in diabetes (Barnosky et al., 2014b). Certainly, more investigation is required to ascertain the efficacy of the intermittent fasting on cardiovascular risk as well as determine if a certain protocol is associated with more benefit.

Significantly, there has been no structured effort to investigate whether any of these positive cardiovascular outcomes of IF were mediated by its impact on AT remodeling independent of other metabolic factors. Indeed, the cardiometabolic markers consistently reported to be improved by IF like serum lipid and insulin levels as well as insulin resistance are those closely linked to AT inflammation. AT depots in immediate contact with the cardiac and vascular tissue microenvironment, namely PVAT and epicardial AT, would be ideal targets to mediate such effects. Limited changes in the status of immune cell and adipokine secretome of these depots could be sufficient to exert profound effects on cardiovascular structure and function. Interestingly, this highlights the importance and potential utility of IF protocols in the cardiovascular outcome of early metabolic impairment where adipose inflammation plays a more important role vs. hyperglycemia being a more predominant factor in more advanced conditions of metabolic decompensation in diabetes (Elkhatib et al., 2019; Bakkar et al., 2020b).

## Conclusion

Adipose tissue deleterious remodeling and CMS are tightly linked to positive energy balance and HFD intake (Bays et al., 2007). PVAT has been recently implicated as one of the integral components of CMS that is highly sensitive to energy imbalance (Almabrouk et al., 2018; Qi et al., 2018). Positive energy balance appears to induce PVAT inflammation preceding other AT depots, which in turn triggers cardiovascular dysfunction early in the course of metabolic impairment. IF alleviates the metabolic and AT derangements known to accompany CMS. As IF promotes beneficial AT remodeling, the inflammatory state is ameliorated with the potential consequence of improved cardiovascular structural and functional status. Whereas the peculiar nature of PVAT and its localization in close proximity to vascular tissue has brought it to the forefront of investigation of CMS, the impact of IF on PVAT under these circumstances is not well-studied. We propose a framework whereby IF relieves PVAT inflammation and thus induces positive cardiovascular outcomes in such a way that makes IF a feasible intervention with the early manifestations of CMS. Indeed, this is supported by the fact that prolonged intermittent fasting elicits pro-longevity metabolic alterations. However, for such an intervention to be of relevance in the treatment or prevention of CMS, the safety of prolonged fasting must be assured (Longo and Panda, 2016). A recent study highlighted that healthy individuals who have undergone alternate day fasting for six months had reduced cholesterol levels, inflammatory markers, and CVD risk (Stekovic et al., 2019). In another study that employed the Buchinger fasting protocol (fasting period between 4 and 21 days), adverse effects were reported in less than 1% of the 1,422 study subjects (Wilhelmi De Toledo et al., 2019). A review of studies following patients for up to twelve months only reported mild headaches as adverse outcomes of intermittent fasting (Allaf et al., 2021). Indeed, this indicates that prolonged alternate day fasting and complete fasting protocols are safe and well tolerated, and provides clinical evidence for their feasible utilization.
